# Photoluminescence emission of nanoporous anodic aluminum oxide films prepared in phosphoric acid

**DOI:** 10.1186/1556-276X-7-689

**Published:** 2012-12-29

**Authors:** Abolghasem Nourmohammadi, Saeid Jalali Asadabadi, Mohammad Hasan Yousefi, Majid Ghasemzadeh

**Affiliations:** 1 Department of Physics, Faculty of Science, University of Isfahan, Isfahan, 81746-73441, Iran; 2 Nanotechnology Research Group, Faculty of Applied Sciences, Malek-Ashtar University of Technology, Shahinshahr, Isfahan, 83145-34177, Iran

**Keywords:** Al_2_O_3_, Anodizing, Phosphoric acid, Oxygen vacancies, Luminescence

## Abstract

The photoluminescence emission of nanoporous anodic aluminum oxide films formed in phosphoric acid is studied in order to explore their defect-based subband electronic structure. Different excitation wavelengths are used to identify most of the details of the subband states. The films are produced under different anodizing conditions to optimize their emission in the visible range. Scanning electron microscopy investigations confirm pore formation in the produced layers. Gaussian analysis of the emission data indicates that subband states change with anodizing parameters, and various point defects can be formed both in the bulk and on the surface of these nanoporous layers during anodizing.

## Background

Porous anodic aluminum oxide (PAAO) is an amorphous type of aluminum oxide, Al_2_O_3_, which is produced via anodic oxidation of aluminum in acidic electrolytes such as sulfuric, phosphoric, and oxalic acid [1]. This nanoporous material is often used as the deposition template for fabricating one-dimensional nanostructured materials because it offers self-organized arrays of the pores in the nanoregion. The geometric coefficients of the pores such as pore size and aspect ratio can be altered over vast areas by varying the anodizing parameters. Therefore, it is possible to produce uniform one-dimensional nanomaterials with different size and aspect ratio by using these templates. Moreover, they offer the advantage of possible *insitu* annealing of the grown nanomaterial because of the thermal stability of aluminum oxide. PAAO films have other useful properties such as optical transparency over a wide spectral range and having low cost.

All the Al_2_O_3_ polymorphs are known as wide-band gap dielectric materials with large band gaps of 7 to 9 eV [2]. Recently, we calculated the band gap of γ-Al_2_O_3_ compound in close agreement with experimental data [3]. The calculations were performed in the framework of the density functional theory as embodied in the reliable WIEN2k code [4] using the modified Becke-Johnson (mBJ) exchange potential [5] by optimizing the c factor of the mBJ method. Large band gaps are assumed for different PAAO layers because the crystal structure of amorphous Al_2_O_3_ is close to the surface structure of γ-Al_2_O_3_ polymorph at room temperature [6]. Thus, very low efficiency of free carrier photogeneration could be attributed to PAAO at room temperature. However, an interesting semiconductor behavior is observed in the barrier layer of PAAO layers which are formed in the phosphoric acid electrolyte [7-10].

As we expect, PAAO materials are insulators at room temperature, but the experimental results show that they have semiconductor behavior. This is just for existence of subband levels in the electronic structure of PAAO layers that enable carrier photogeneration at room temperature. Here, the PL properties of PAAO films formed in phosphoric acid are investigated under different anodizing conditions in order to identify the subband levels in these materials. Many detailed studies are reported in the related literature for the determination of the electronic structure of PAAO layers anodized in oxalic acid via the PL properties [11-15] because the strongest PL activity exists in these nanoporous layers. There are few articles reporting the optical properties of PAAO layers formed in different electrolytes including phosphoric acid [16,17]. However, they have emphasized on the contribution of the type of the electrolyte, and no mention about the effect of anodizing condition on the PL properties of the anodic films formed in the phosphoric acid electrolyte. This topic is studied by us in detail.

## Main text

The first part of this study is to prepare PAAO membranes through two-step anodization of high purity (99.997%, Alfa Aesar, Karlsruhe, Germany). First of all, aluminum foils are cleaned in ethanol and acetone in sequence using ultrasonic vibration, and the foil surfaces are chemically cleaned in a mixture of HCl, HNO_3_, and H_2_O with molar ratios of 10:20:70, respectively. To improve the pore order, the aluminum foils are first annealed in ambient nitrogen at 500°C to increase the aluminum grain size and reduce their internal grain boundaries in order to achieve long-range homogeneity in the foils. Then, the aluminum foil surfaces are electrochemically polished using a mixture of H_3_PO_4_, H_2_SO_4_, and H_2_O with 4:4:2 weight ratios, respectively [18]. As reported in [7,8], this process can decrease foil surface roughness down to submicron scales and remove the surface imperfections which are present on the aluminum foil after its rolling. The anodizing is carried out in a homemade anodizing cell cooled down to 2°C using high purity phosphoric acid as the electrolyte (85 wt.%, Merck, KGaA, Darmstadt, Germany). The foil temperature is kept constant at 1°C. Various anodizing voltage and time are used. After anodizing, the remaining Al substrate is etched away in a saturate solution of HgCl_2_ at room temperature in order to achieve transparent aluminum oxide membranes.

A VEGA- TESCAN scanning electron microscope (SEM) system (Brno, Czech Republic) is employed to confirm pore formation in the anodic layers and study size and morphology of the membrane pores. The PL spectral measurements are carried out on a PL spectroscopy LS55 system (PerkinElmer Inc., MA, USA) equipped with a Xe lamp as the light source. The PL results are Gaussian fitted, using the ‘Peak Fitter Toolbox’ in Matlab software (The MathWorks, Inc., MA, USA), in order to investigate quantitatively the effect of the anodizing parameters on the PL emissions and display formation of different point defects in the prepared membranes.

## Discussion

### SEM analysis

**Figure 1:**
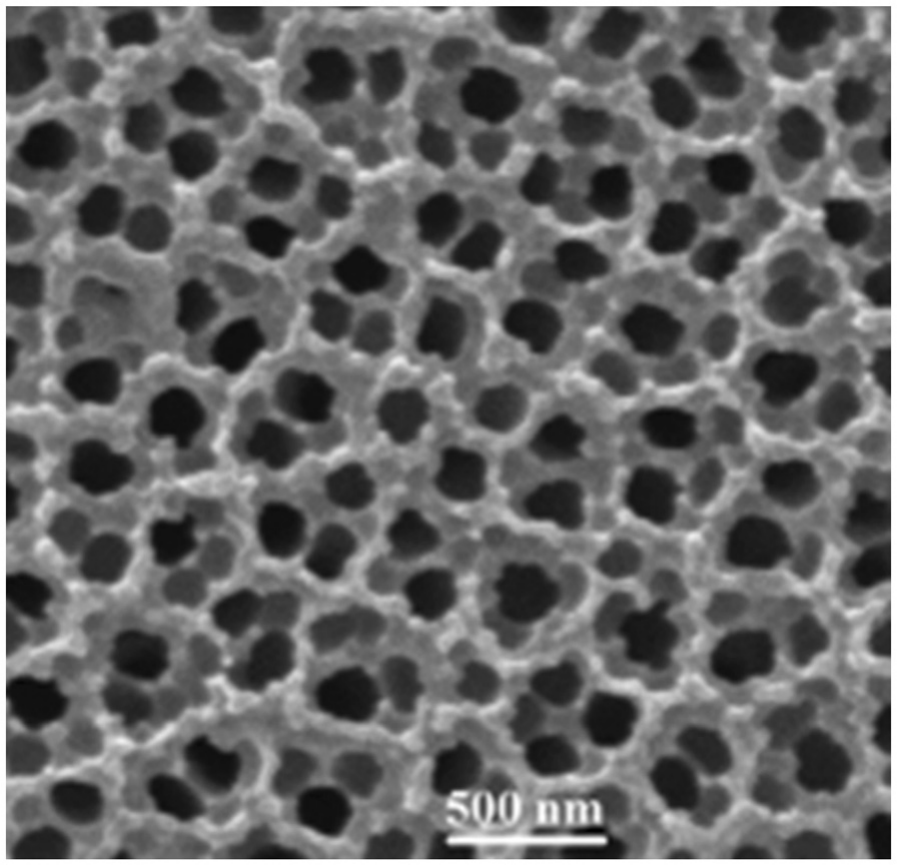
SEM planar view of an anodic alumina membrane anodized at 130 V.

A typical SEM planar view of a PAAO membrane, prepared as described above, is illustrated in Figure 1. This membrane is anodized at 130 V for 20 h in the phosphoric acid solution. Since both sides of the prepared membranes are etched in a saturate HgCl_2_ solution, partial etching of the membrane pores is occurred. As a result, the morphology of the membrane pores is disordered, and the pore internal diameters appear different (see Figure 1). However, the average pore center-to-center distance is measured to be about 230 nm.

### Effect of applied voltage

To evaluate the effect of anodizing voltage, both the first and the second anodizing steps are carried out by applying similar DC voltages ranging from 100 to 130 V for fix anodizing time of 20 h. This range of voltages is selected based on our previous observation on the optimized semiconductor activity of the PAAO membranes formed via aluminum anodizing at approximately 115 V for up to about 20 h [10].

Different excitation wavelengths are tested in order to identify most of the details of the subband states. It is observed that under 265-nm excitation wavelength, the PL emission includes most of the emission peaks which are observed by exciting the membranes under different excitation wavelengths solely. Hence, our interpretation of the defect-based subband states is based on the PL emissions measured under 265-nm excitation.

**Figure 2:**
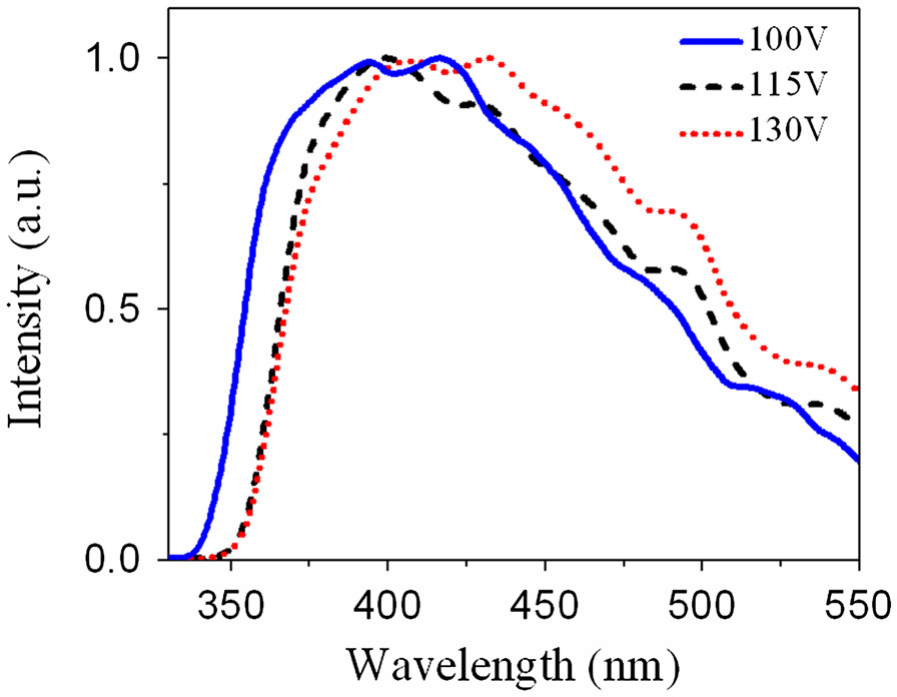
PL emission spectra of PAAO membranes formed, using different anodizing voltages, in phosphoric acid.

All the measured PL emission spectra of the membranes produced at 100, 115, and 130 V, are presented in Figure 2. It is observed that all the membranes show PL emission in the 300- to 550-nm wavelength range. Qualitatively, a redshift is observed within some of the measured PL spectra (see Figure 2). It is evident that an increase in anodizing voltage leads to a slight shift in the emission peaks toward the visible region. Thus, the subband gaps present in the electronic structure of the membranes are narrowed slightly by an increase in anodizing voltage. It should be pointed out that the shift rate is much more below 115 V, and it decreases afterward. It could be deduced that in these membranes, an increase in anodizing voltage by approximately 115V enhances formation of optically active defects with subband gaps which lay in the visible range.

The PL emission of metal oxides usually has various origins like intrinsic electronic point defects. It is known that for isolated similar point defects in an amorphous material, the PL emission has a normal (Gaussian) shaped distribution. In the case of different light-emitting point defects, the PL emission regarding each defect type will contribute to the whole emission spectrum through a Gaussian-like peak. Gaussian fitting analyzes these contributions and assists us to identify different electronic point defects which arise in the PAAO membranes.

**Figure 3:**
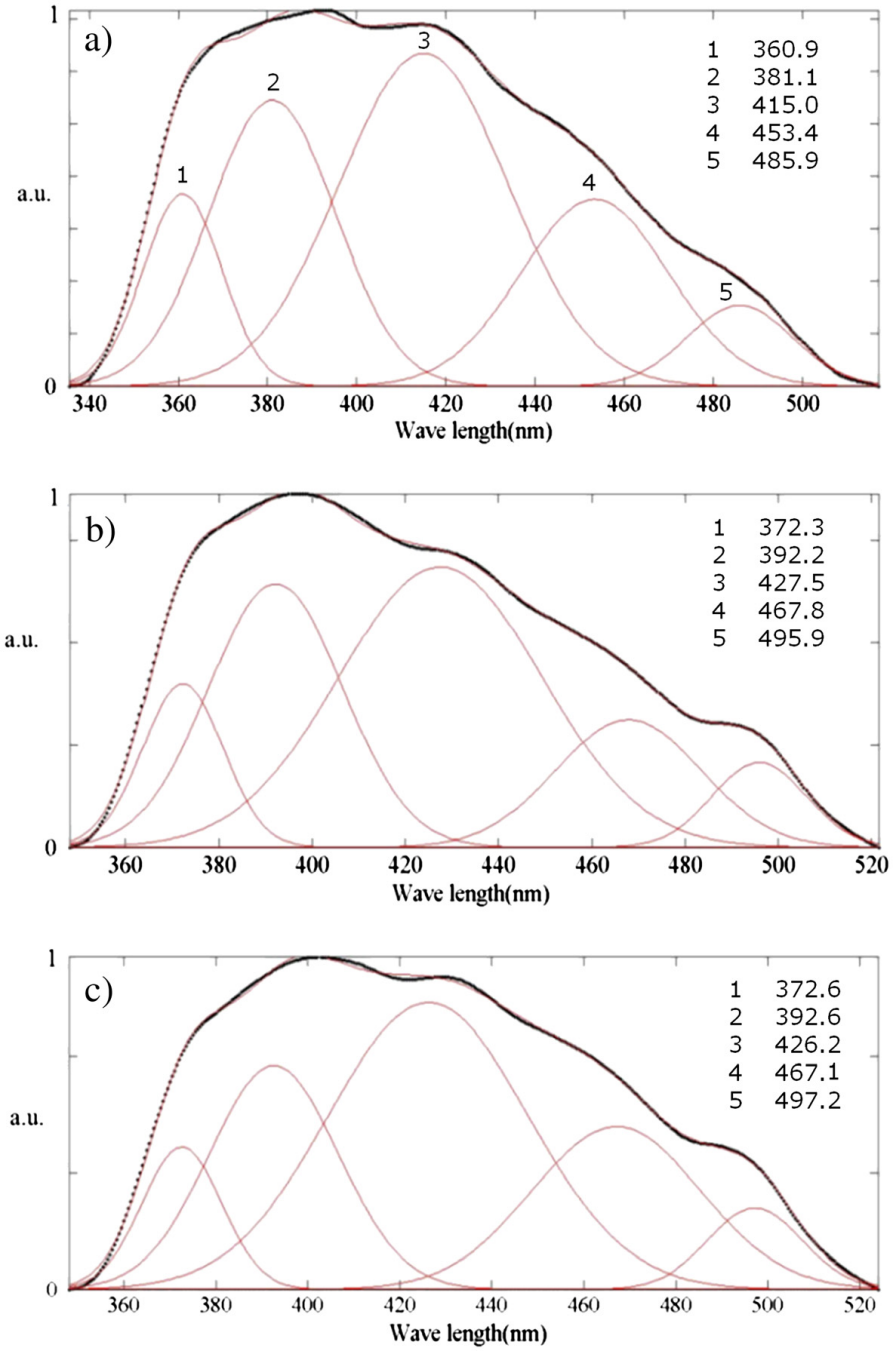
**Fitted PL emission spectra of the aluminum oxide membranes of Figure**2**.** The membranes are anodized at (**a**) 100, (**b**) 115, and (**c**) 130 V.

The analyzed emission spectra of Figure 2 are shown in Figure 3a,b,c. Those figures show that PL emission of all the membranes are composed of five different Gaussian-shaped functions. The Gaussian functions in Figure 3a are fitted to peaks about 361, 381, 415, 453, and 486 nm which correspond to 3.43, 3.25, 2.99, 2.74, and 2.55 eV subband transitions, respectively. But these transitions are fitted out at 372, 392, 428, 468, and 496, and at 373, 393, 426, 467, and 497 nm wavelengths, which have energies of 3.33, 3.16, 2.90, 2.65, and 2.5, and of 3.32, 3.15, 2.91, 2.65, 2.49 eV, respectively (see Figure 3b,c). These results show that an increase in anodizing voltage from 100 to 115 V leads a rather equal amount of redshift in the position of all the PL emissions, see for instance peaks 1 and 2 in Figure 3a,b.

In Figure 3a, the 415-nm peak reveals the maximum emission intensity. This emission wavelength is close to the beginning of the blue region. However, the maximum emission locates about 427 nm in Figure 3b,c, which is close to the middle of the blue region. This wavelength shift can slightly improve the PL activity of the membranes in the visible range.

In Figure 3c, peak positions show negligible shift compared with Figure 3b. A tolerance error should be considered for both PL measurement and graph fitting procedures because the fluorescence spectrophotometer precision lies at approximately 0.1 nm, and there exists a possibility of error in the fitting process. Consequently, it could be deduced that an increase in the anodizing voltage beyond 115 V has insignificant shifting effect on the emission spectrum (see Figure 3b,c). These findings indicate that an increase in the anodizing voltage beyond 115 V cannot enhance the PL activity of the membranes in the visible range.

**Figure 4:**
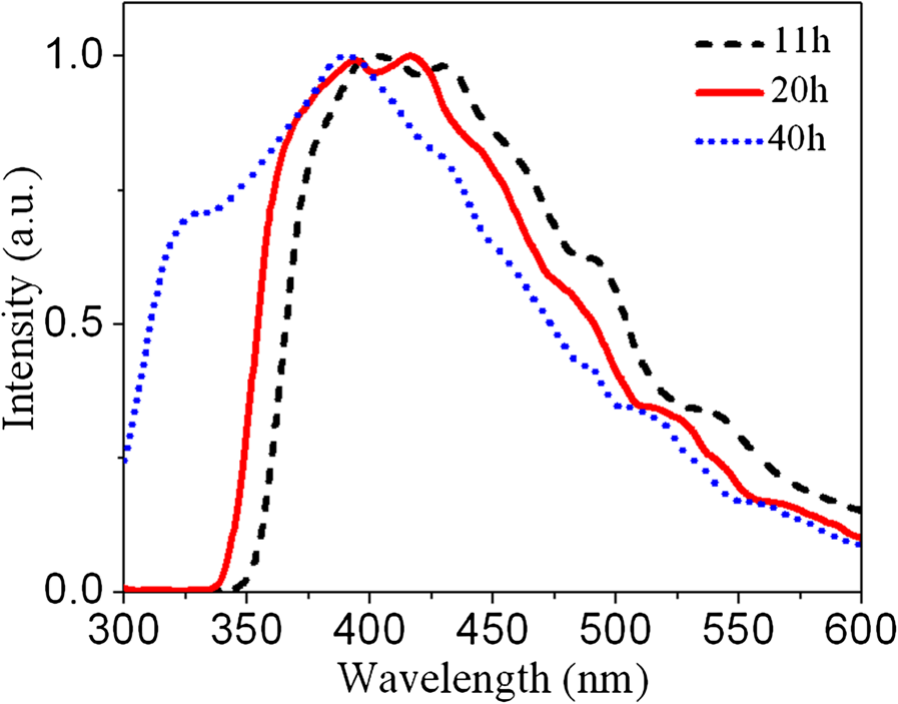
Dependence of the PL emission spectra to the anodizing time.

Most of the previous reports have related the PL properties of PAAO layers to the optical transitions within individual oxygen vacancies. However, there is a clear-cut distinction between their interpretations on the type of the oxygen vacancies. Some researches claim in their articles that the PL spectra are concerned to the singly ionized oxygen vacancies [12,13,15]. But others relate the spectra to both singly ionized and neural oxygen vacancies [11,14]. Singly ionized oxygen vacancies are generally called F^+^ centers. These point defects form when an electron is trapped in a double ionized oxygen vacancy. Neutral oxygen vacancies are often called F centers. They can be formed if two electrons are trapped in a double ionized oxygen vacancy. Our results could not confirm the interpretations of the first group; otherwise, our results would not agree with the results on crystalline Al_2_O_3_. According to Lee and Crawford studies on sapphire [19] and Evans and coworkers on crystalline α-Al_2_O_3_[20], if crystalline Al_2_O_3_ is excited under a 4.8 eV (260 nm) wavelength, it would emit UV PL radiation due to the F^+^ color centers at approximately 3.8 eV (326 nm). Only one PL emission about 3.8 eV is fitted out among our results (see the 323-nm peak in Figure 4c). But several visible emissions far greater than 323 nm are identified (Figure 3a). As it is discussed in the next section, our results correspond better with the interpretations of some of the second group of researchers who suggest that F^+^ centers exist in the bulk structure of PAAO membranes, and neural oxygen vacancies, F centers, are on their surface [11]. Chen and coworkers [21] report measurement of a blue PL emission approximately 420 nm in sapphire due to F^+^ color centers using a 244-nm excitation wavelength. This excitation is close to the optimized excitation wavelength identified in our study, 265 nm, and several emissions around 420 nm are fitted out in our analyzed PL data (see Figure 3a,b,c). It is shown in the next section that most of these emissions originate from bulk of the nanoporous layer, and emissions which are far greater than 323 nm are from the layer surface.

As a summary, it could be said that in PAAO membranes anodized in phosphoric acid, the electronic subband gaps due to oxygen vacancies can be altered by the anodizing voltage; an increase in anodizing voltage up to 115 V narrows the electronic subband gaps, and beyond 115 V, no sensible effect is observed. These results may be helpful in explaining our previous results on optimization of the room-temperature semiconductor behavior of the nanoporous layers anodized under about 115 V [10].

### Effect of anodizing time

To evaluate anodizing time effect, the PL wavelength spectra of the PAAO membranes anodized at 100 V for 11, 20, and 40 h are measured, as shown in Figure 4. All the spectra of Figure 4 are obtained at 265-nm excitation wavelength in order to study most of the optical transitions. This figure indicates that an increase in the anodizing time can both widen the whole emission spectrum of the membranes and shift it toward shorter wavelengths in a qualitative manner. Significant widening and shifting toward UV region are observed for 40-h anodizing time. Thus, an increase in anodizing time by 40 h aids formation of the optically active oxygen vacancies with subband gaps which are out of the visible range. This phenomenon reduces the emission activity of the PAAO membranes in the visible region.

**Figure 5:**
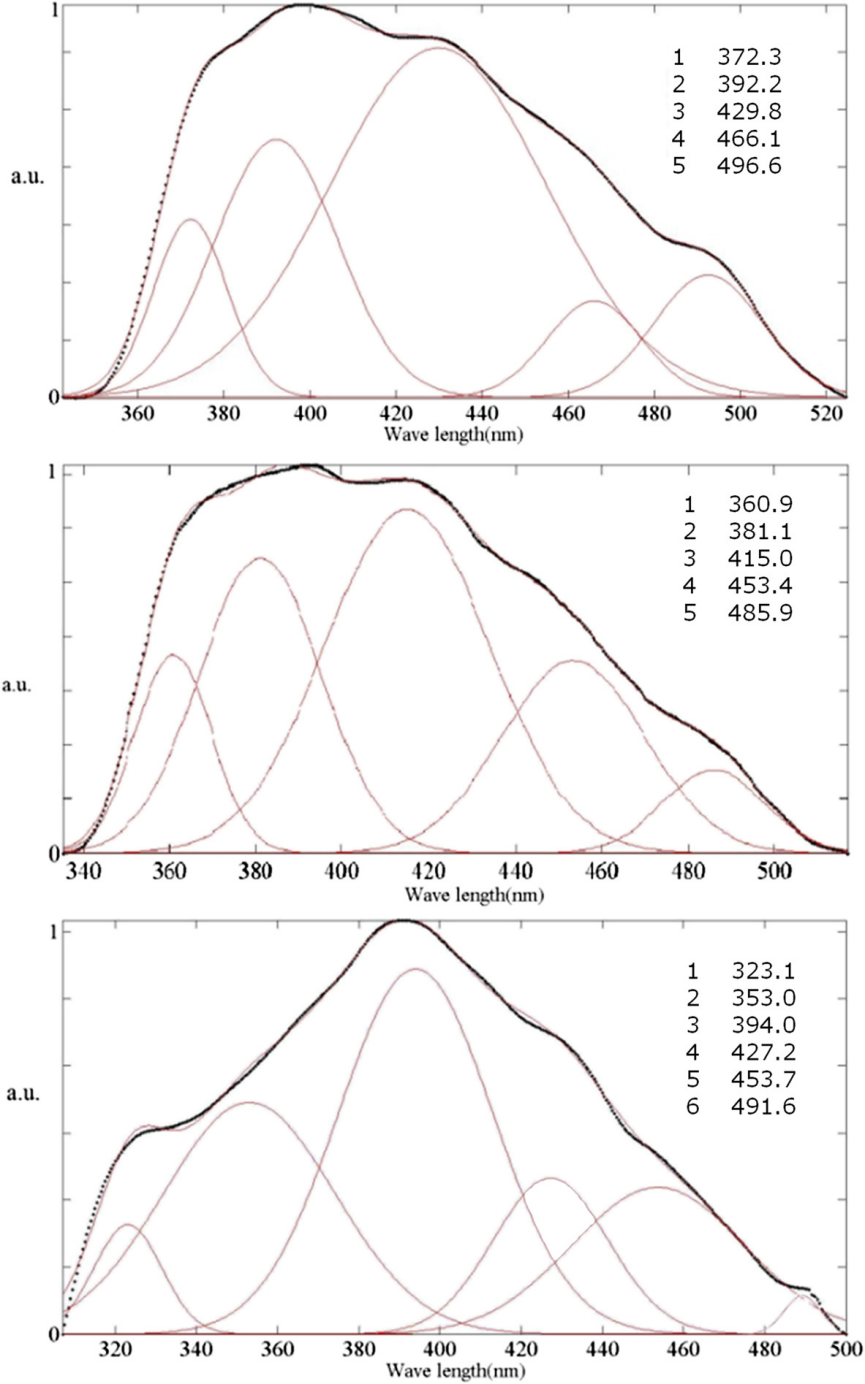
**Fitted photoluminescence emissions of the PAAO membranes.** The membranes were prepared after (**a**) 11, (**b**) 20, and (**c**) 40 h of anodizing.

Different PL emissions of the samples of Figure 4 are analyzed in Figure 5 in order to evaluate the effect of anodizing time on the subband transitions quantitatively. The analyzed emission spectra of the membranes anodized at 100 V over 11- and 20-h time periods are shown in Figure 5a,b, respectively. Both spectra are composed of five contributive peaks. In Figure 5b, the same emission spectrum of Figure 3a is shown in order to compare the effect of the anodizing time on the subband transitions. The position of all Gaussian emissions of Figure 5b show a rather equal blueshift compared to the membrane of Figure 5a (see for instance peaks 1 and 2 in both figures). In Figure 5a, the maximum emission intensity takes place about 430 nm, which is close to the middle of the blue region. However, the maximum emission intensity of Figure 5b is at 415 nm which is close to the beginning of the blue region. For the membrane which is anodized for 40 h (Figure 5c), a high emission peak is observed at 394 nm which is quite close to the ultraviolet region. This confirms quantitatively widening of the electronic subband gaps due to the oxygen vacancies during a longtime anodizing process.

Some pioneering but advanced studies on PAAO layers have shown that after formation of the pores, a steady state regime of pore growth occurs [1]. In this regime, the porous Al_2_O_3_ layer thickens with time, and no principal evolution occurs in its morphology. It might be deduced that an increase in the anodizing time would only increase the PL line intensities. However, a considerable blueshift is observed in all the PL emissions with an increase in the anodizing time (see Figure 5). This shift points out that time period of voltage application can affect the subband electronic gaps in the anodic oxide layer.

According to Huang and coworkers [11], F+ centers distribute mainly in the bulk structure of the PAAO layers and F centers are mainly on their surface. The anodizing electric field will drift the anions suspended in the electrolyte toward the anode (i.e., PAAO layer). Therefore, during voltage application, surface double charged oxygen vacancies can trap easily two electrons from the negatively charged anions to become neutral (F center). Our findings may confirm this argument. While the PL spectrum is gradually widened with increasing anodizing time from 11 to 40 h, the relative intensity of the first three peaks is not appreciably changed (see peaks 1 to 3 in Figure 5a,b,c). It can be deduced that these emissions originate from F^+^ centers which arise in the bulk of the amorphous PAAO layers during anodization in phosphoric acid.

An increase in the anodizing time from 11 to 20 h has reversed the relative intensity of the last two peaks (see peaks 4 and 5 of Figure 5a,b). Besides, the relative intensity of these two peaks is changed again after 40-h anodizing, as can be seen in Figure 5c. It can be concluded that those emissions originate from surface oxygen vacancies. Both of the mentioned emissions lay within the visible range (Figure 5). The presence of narrow band gap F centers on the surface may help us explain the semiconductor behavior of PAAO films at room temperature.

The Gaussian analysis shows that after a short anodizing time, the PL emissions are composed of five Gaussian functions (see Figure 5a,b). On the contrary, after a long anodizing, the PL spectrum has six Gaussian contributions, and an extra Gaussian emission is observed about 492 nm (within the blue-green border); see Figure 5c. This difference could be due to formation of a different-type PL emitting origin, likely an ensemble of surface oxygen vacancies, after applying voltage for a long time.

## Conclusions

Porous anodic aluminum oxide films are successfully prepared via two-step anodization of high purity aluminum foils in phosphoric acid, and their electronic subband structure is studied via their PL emission. It is found that both the anodizing voltage and time can affect the PL emissions of the produced layers. An increase in anodizing voltage between 100 to 115 V leads to a redshift in the PL emissions and improves the PL activity of the layers in the visible region. It means that the defect-based subband gaps present in the prepared layers are narrowed. An increase in the anodizing time between 10 to 40 h shifts the PL emissions spectra toward the ultraviolet region and creates new point defects. This effect widens the defect-based subband gaps and decreases their PL activity in the visible range. Our results show that anodizing parameters that optimize the PL activity of the nanoporous layers in the visible range are close to those which optimize the semiconductor behavior of the layers at room temperature. Therefore, PL investigations could be helpful in explaining this semiconductor behavior. Most of the Al_2_O_3_ polymorphs exhibit good thermal and chemical stability and, depending on their specific properties, are used in a variety of applications. The semiconductor behavior of this type of Al_2_O_3_ makes PAAO a promising material for future applications.

## Competing interests

The authors declare that they have started the process of patent application in the US patent office relating to the content of this manuscript. The authors will ask Iran Nanotechnology Initiative Council and Chemnitz University of Technology in Chemnitz, Germany for financial support for patent application fees.

## Authors’ contributions

AN is the director of this experimental study and has drafted this manuscript. MG, as a MSc student, is jointly supervised by SJA to simulate the compound in question, as discussed in [3] and background sections of this paper, and by AN to carry out the experimental measurements, as discussed in this paper. MHY participated in the experimental studies by PL measurements. All authors read and approved the final manuscript.

## Authors’ information

Dr. AN is an assistant professor of experimental condensed matter physics at the Department of Physics, University of Isfahan, Isfahan, Iran. His research interests cover oxide and II-VI semiconductors, soft magnetic materials, and ferroelectrics. Dr. SJA is an assistant professor of computational condensed matter physics at the Department of Physics, University of Isfahan, Isfahan, Iran. Dr. SJA is interested in performing density functional theory-based ab initio calculations to study electronic, structural, hyperfine interactions including magnetic hyperfine fields and electric field gradients, quantum size effects, acoustic, and optical properties of a broad range of materials including strongly correlated systems and biomaterials in bulk, surface, interface, nanowire, and quantum dot forms. Dr. MHY is an associate professor of Nanotechnology Research Group, Faculty of Applied Sciences, Malek-Ashtar University of Technology, Shahinshahr, Isfahan, Iran. His research interests are nanomagnetism, II-VI quantum dots, and nanowires.
